# Enhanced Recovery after Surgery for Lung Cancer Patients

**DOI:** 10.1515/med-2020-0029

**Published:** 2020-03-19

**Authors:** Feng Chen, Gongchao Wang

**Affiliations:** 1 Shandong University, 44 Wenhua West Road, Jinan 250012, China; 2 Thoracic surgery ward,Shandong Cancer, Hospital and Institute, Shandong First Medical University and Shandong Academy of Medical Sciences, Jinan 250117, P .R.China

**Keywords:** Lung cancer, Enhanced recovery after surgery, Nursing

## Abstract

**Background:**

Enhanced recovery after surgery (ERAS) is a perioperative treatment intended to speed up recovery of surgical patients. Pulmonary lobectomy is a high-risk procedure, which ERAS is intended to address.

**Objective:**

We evaluated the application of ERAS to patients with lung cancer.

**Interventions/Methods:**

Of 337 patients who underwent pulmonary lobectomies for lung cancer at our hospital, 168 received traditional perioperative nursing, and 169 received ERAS. Their complication rates, numerical rating scale of pain (NRS), satisfaction with care, demographics and some inpatient indices before and after surgery were compared.

**Results:**

The two groups did not significantly differ in general data or NRS score at 6 post-operative hours (P = 0.214) and 1 post-operative day (POD; P = 0.027). The ERAS group had lower incidence of postoperative lung complication (P = 0.008), shorter length of stay (P < 0.001), shorter enterokinesia recovery times (P < 0.001), lower hospitalization costs (P < 0.001), lower NRS scores at POD 2–5 (P < 0.001), higher nursing satisfaction (P < 0.001), and higher postoperative pulmonary function indices of maximal voluntary ventilation (MVV; P < 0.001), forced vital capacity (FVC; P = 0.002), and forced expiratory volume in 1 second (FEV1; P = 0.002) than did the traditional group.

**Conclusion:**

ERAS is applicable to lung surgery patients.

**Implications for Practice:**

Applying ERAS to thoracic surgery patients can alleviate their postoperative pain, shorten enterokinesia recovery, lower postoperative complication incidence, reduce hospitalization expenses, and raise nursing satisfaction, thereby expediting recovery.

## 1 Introduction

Enhanced recovery after surgery (ERAS) is a recent approach to perioperative treatment intended to speed recovery, by reducing postoperative complications and alleviate stress of surgical patients [1]. The idea was first proposed by Henrik Kehlet, a Danish professor of surgery. Its central idea is to use effective perioperative managements confirmed by evidence-based medicine to minimize the stress response of the operation to the body, so as to shorten the postoperative hospital stay, reduce hospitalization expenses, and speed the recovery of the postoperative surgical patients. Pulmonary lobectomy is a high-risk procedure with surgical trauma, high incidence of postoperative complications, and high hospitalization expenses, which are some outcomes ERAS is intended to address [2].

In September 2016, our department adopted ERAS measures for perioperative nursing, included four steps (a) admission education (b) respiratory function exercises (c) analgesia management (d) early postoperative ambulation.

A retrospective analysis was made on the clinical data of traditional group and intervention group, included the incidence of postoperative complications, postoperative hospital stay, postoperative pain score, bowel sound recovery time, and nursing satisfaction index. Enhanced recovery after surgery (ERAS) among thoracic surgery patients have obtained satisfactory results, which was clinically conducive to the popularization of the concept of change. Meanwhile, it could provide the clinical basis for the confirmation of the standardization process. It is reported as follows.

## 2 Methods

### 2.1 Patients

We selected 337 patients who had undergone pulmonary lobectomies for lung cancer from July 2015 to June 2017 in our thoracic surgery department with the use of convenient sampling. Among these patients, 168 patients received conventional treatment during their perioperative periods, and 169 patients (treated after September 2016) received ERAS treatment. We included patients who (a) had resectable stage I–III lung cancer; (b) received no radiotherapy or chemotherapy before surgery; (c) showed no involved lymph nodes in the pulmonary hilus, mediastinum or other malignant tumors on preoperative CT examination; and (d) had no major organ lesions or suffered from liver or kidney dysfunction. We excluded patients who had undergone exploratory surgery or multilobe excision; or who had benign lung lesions, autoimmune disease, or cognitive disorders.

Patients gave informed written consent to ERAS treatment, which had also been approved by the hospital’s ethics committee. Six patients were excluded from exploratory surgery or polylobectomy: five were excluded for benign lung lesions, one was excluded for autoimmune disease, and one was excluded for cognitive impairment.

## 3 Treatment

The conventionally treated group received (a) admission education, including information on medical history taking, ward environment, visiting system, security, lead medical personnel, diet and their surgeries; (b) respiratory function exercises, including lip-constriction abdominal respiration method and blowing-ball breathing method; (c) analgesia management that included 75–100 mg of pethidine hydrochloride injected intramuscularly for those whose pain-rating scale (NRS) scores were ≥4, but no preventive analgesic after surgery; and (d) early postoperative ambulation during which patients had bedrest on post-operative day (POD) 1, received bedside radiography, had their catheters removed, and were assisted in expectoration by tapping their backs on POD 2, and had chest tube removed (depending on the chest radiograph results) before being allowed gradual ambulation on POD 3.

The ERAS group received (a) admission education, including brochures on enhanced recovery from their receiving physicians, a 2-hour lecture and slide show (given each Tuesday and Friday by the lead and charge nurses), and encouragement to participate from the lead nurse, who was a qualified psychological consultant; (b) respiratory function training, including 4–6 daily training sessions of 10–15 minutes each, starting before surgery and restarting on POD 1, depending on the patient’s strength and other circumstances; (c) preventative analgesia management, including 5 mg of oral oxycodone and acetaminophen tablets 3× daily starting on POD 1, and replaced with other drugs if the patient showed symptoms such as dizziness, constipation, nausea, vomiting; and (d) early ambulation, including deep breathing and coughing after waking up on POD 2, changing bed posture 6 hours after surgery, sitting on the bedside, then standing and finally walking on the morning of POD 2, under the nurses’ observation and subject to the patient’s physical limitations. The comparison between the two groups is shown in the table below.

### 3.1 Observational indices

By consulting patient and departmental records, we observed and compared numbers of PODs in hospital, hospitalization expenses, incidence of postoperative complications, borborygmus recovery time, postoperative NRS, nursing satisfaction and changes in major pulmonary function indices MVV, FVC, FEV1 on admission and before surgery for both groups. We used NRS to measure pain degree and nursing job satisfaction questionnaire to measure satisfaction degree toward nursing.

### 3.2 Statistical analysis

All the data were analyzed using SPSS 17.0 software, and expressed as x±s or as n (%). We used Student’s t test to compare mean values, and the chi-square test to compare rates. α=0.05 was considered significant.

## 4 Results

### 4.1 General patient data

The two patient groups did not significantly differ in sex (P = 0.968), age (P = 0.719), basic diseases which consisted of 3 illnesses (P = 0.839), surgical sites and procedures which consisted of 5 subcategories (P = 0.056), pathology which included squamous carcinoma and adenocarcinoma (P = 0.162), and clinical TNM staging included 5 subcategories (P = 0.880).

**Table 1:** Comparison of Perioperative Nursing Measures

	Group	From hospital admission to surgery	From surgery to discharge
Admission education	ERAS group	a) brochures about ERAS on admission b) lecture and slide show about ERAS were given twice a week c) encouragement from lead nurse to along with treatment	-
	Control group	routine nursing	-
Respiratory function training	ERAS group	respiratory function trainer	respiratory function trainer
	Control group	-	lip-constriction abdominal respiration or blowing-ball breathing
	ERAS group	-	preventative analgesia management,
	Control group	-	analgesic injected when NRS scores were ≥4
Pain management Off-bed activity	ERAS group	-	POH 6: change bed posture POD 2: standing and walking under nurses’ observation
	Control group	-	POD 1: bedrest POD2: bedside radiography, catheters removed POD3: chest tube removed, gradual ambulation

**Table 2:** Comparison of the patients’ general data

Item	Content	Group A (n=168)	Group A (n=169)	р
Age	Year	57.22±8.95	57.60±10.23	0.719
Sex	Male	109(64.9%)	110(65.1%)	0.968
	Female	59(35.1%)	59(34.9%)	
Pathology	Adenocarcinoma	100(59.5%)	113(66.9%)	0.162
	Squamous carcinoma	68(41.5%)	56(33.1%)	
	Hypertension	34(20.3%)	28(18.3%)	
Underlying diseases	Coronary heart disease	16(9.6%)	21(13.7%)	0.839
	Diabetes	21(12.5%)	18(11.7%)	
	Superior lobe of right lung	45(26.8%)	61(36.1%)	
	Middle lobe of right lung	12(7.1%)	11(6.5%)	
Lesion site	Inferior lobe of right lung	45(26.8%)	34(20.1%)	0.056
	Superior lobe of left lung	32(19.0%)	43(25.4%)	
	Inferior lobe of left lung	34(20.2%)	20(11.8%)	
Pathological staging	ⅠA	36(21.4%)	34(20.2%)	0.880
	ⅠB	43(25.5%)	39(23.1%)	
	ⅡA	30(17.9%)	37(21.9%)	
	ⅡB	32(19.1%)	30(17.6%)	
	ⅢA	27(16.1%)	29(17.2%)	

### 4.2 Clinical outcomes

The ERAS group had significantly shorter postoperative borborygmus recovery time and hospitalization times, less hospitalization expense and lower incidence of postoperative atelectasis and lung infection, and improved preoperative pulmonary function indices, higher nursing satisfaction scores and NRS scores at PODs 2–5 than did the conventionally treated patients. The groups’ NRS scores at 6 hours and 1 POD, and incidences of postoperative arrhythmia did not significantly differ (Tables 3–6).

**Table 3:** The comparison of the recovery time of borborygmus, postoperative hospitalization time, hospitalization expenses, and nursing satisfaction between the two groups

Item	Unit	Group A (n=168)	Group A (n=169)	P value
Recovery time of borborygmus	Hour/h	26.12±3.34	21.40±2.60	<0.001
Postoperative hospitalization time	Day/d	11.98±4.00	8.91±2.43	<0.001
Hospitalization expenses	Ten thousand Yuan	4.96±1.22	4.36±0.76	<0.001
Nursing satisfaction Comprehensive score	Point	141.30±2.62	143.08±2.03	<0.001

**Table 4:** Comparison of incidence of postoperative complications between the two groups of patients

Postoperative complication	Group A (n=168)		Group A (n=169)		P value
	Case load	Incidence	Case load	Incidence	
Atelectasis	11	6.59%	3	1.78%	0.030
Lung infection	15	8.92%	5	2.96%	0.020
Arrhythmia	6	3.57%	3	1.78%	0.248
Total	32	19.04%	11	6.51%	0.008

**Table 5:** Comparison of postoperative NRS between the two groups of patients

Category	Group A (n=168)	Group A (n=169)	P value
The sixth hour after surgery	6.02±0.71	5.92±0.70	0.214
Postoperative 1d	5.88±0.63	5.72±0.64	0.027
Postoperative 2d	4.92±0.86	3.64±0.78	<0.001
Postoperative 3d	3.81±0.65	2.73±0.72	<0.001
Postoperative 4d	3.29±0.75	2.37±0.59	<0.001
Postoperative 5d	2.75±0.67	1.87±0.59	<0.001

**Table 6:** Comparison of pulmonary function indices of the enhanced recovery group in admission and before the surgery.

Item	Unit	Admission	Before surgery	P value
		(n=169)	(n=169)	
MVV (Maximalminute ventilation volume)	L/min	111.13±12.71	131.00±10.85	<0.001
FVC (Forced vital capacity）	L	3.51±0.51	3.66±0.40	0.002
FEV1(Forced expiratory volume in first second ）	L	2.85±0.39	2.98±0.33	0.002

## 5 Discussion

Enhanced Recovery after Surgery suggested by the Danish surgical professor, Henrik Kehlet, and prototypically investigated with colorectal surgery [3] in China, ERAS was first used in 2007 for colorectum cancer surgery at the General Hospital of Nanjing Military Region, from which the first clinical literature on ERAS for gastrectomy was

published [4]. ERAS has been more widely used for chest surgery in recent years. This study found that patients treated with an ERAS approach outperformed the conventional recovery group in all indices before and after the surgery, which is consistent with the findings of Luo Xi, et al [5].

ERAS had several differences from conventional nursing. First, keeping good care of communication is the key to accelerating the rehabilitation concept of successful surgical practice [6]. Directly after diagnosis, patients received detailed explanations of what to expect of their surgeries and treatments, including a brochure and lecture with slide-show. Twice a week of intensive teaching mainly in the form of slides and vivid interpretation of the important items, help those in the low socio-cultural level and the elderly patients to understand and accept the concept. Meanwhile, primary nurses should inform the psychological counseling nurses as soon as they found the abnormal mental states of patients or their relatives. Timely psychological intervention could make patients establish a good psychological state, and actively cooperate with surgical treatment, so as to shorten the length of hospitalization, as well as improving the nursing satisfaction of patients. Secondly, preoperative breathing function exercises were greatly enhanced. After thoracotomy, patients with lung cancer are prone to using an inappropriate breathing mode due to pain, and may have difficulty in coughing, which can lead to such complications as pneumonia and atelectasis [7]. The recommended deep and slow post-surgical breathing mode promotes atelectasis, and increases peripheral airway flow, thus facilitating venting of secretions and reducing the incidence of postoperative lung complications [8]. The compact breathing trainer is easy to carry and has a clear scale indicator and capacity setting that can help the nurse monitor patients and give appropriate feedback. Furthermore, during the study, patients were observed to be more likely to use respiratory trainers. Thirdly, ERAS treatment in our hospital uses an opiate component drug (oxycodone) and a drug recommended by WHO’s three-step analgesia (acetaminophen) tablet to control pain. Records of patients’ postoperative pain indicate that residual anesthetic and patient-controlled analgesia (PCA) pump made the pain tolerable within 1 h and 1 d after surgery, so the NRS score did not significantly change. The NRS score of the ERAS group was significantly reduced at PODs 2–5, which was consistent with the findings of Pavelescu et al. [9] and Renghi et al. [10]. Patients began ambulation at PODs 2–5, a period with a high incidence of pain. Pain can lead to anxiety, restlessness and decreased activity endurance in patients, thus making patients afraid to take effective deep breathing and cough actions, leading to pneumonia, atelectasis and other complications. Use of preventive analgesics can largely alleviate patients’ pain and anxiety, and thus allow them to focus on better breathing and early ambulation. At the same time, timely analgesia can relieve the patient’s tense mood of anxiety, maintain mental stability, help to establish a rehabilitation confidence, and make patients actively cooperate with postoperative nursing intervention measures. Fourthly, out of concern for their safety, conventionally treated patients generally began to ambulate late after the surgery, whereas the ERAS patients were encouraged to move and walk as early as possible. Borborygmus recovery time was obviously shorter in the ERAS group, and early ambulation did not raise the incidence of arrhythmia, which is consistent with the findings of Tiefenthal et al. [11] and Kirk et al. [12]. Early ambulation can promote recovery of intestinal function, significantly shorten the recovery time of intestinal peristalsis, reduce the occurrence of abdominal distension and constipation, so as to enhance nutrition and improve the body resistance. Early activities can also promote lower extremity blood reflux and reduce the occurrence of lower extremity venous thrombosis. Futhermore, early ambulation can improve patients’ breathing capacity and the venting of respiratory secretions, thereby promoting the pulmonary expansion and reducing the incidence of postoperative lung complications [13]. This also enhances patients’ independence and mood, and reduces the family’s burden and pressure.

## 6 Conclusion

The application of ERAS to thoracic surgery can reduce patients’ stress reactions, and postoperative pain, shorten enterokinesia recovery, promote pulmonary expansion, reduce incidence of postoperative complications, decrease hospitalization expenses, and improve nursing satisfaction, thereby expediting recovery; it is therefore both cost-effective and humane.
